# An updated catalog of *CTCF* variants associated with neurodevelopmental disorder phenotypes

**DOI:** 10.3389/fnmol.2023.1185796

**Published:** 2023-05-31

**Authors:** Emma Price, Liron M. Fedida, Elena M. Pugacheva, Yon J. Ji, Dmitri Loukinov, Victor V. Lobanenkov

**Affiliations:** Molecular Pathology Section, Laboratory of Immunogenetics, National Institute of Allergy and Infectious Diseases, National Institutes of Health, Bethesda, MD, United States

**Keywords:** *CTCF*, variant, next-generation sequencing (NGS), mutation, neurodevelopmental disorders, genotype-phenotype

## Abstract

**Introduction:**

*CTCF*-related disorder (CRD) is a neurodevelopmental disorder (NDD) caused by monoallelic pathogenic variants in *CTCF*. The first *CTCF* variants in CRD cases were documented in 2013. To date, 76 *CTCF* variants have been further described in the literature. In recent years, due to the increased application of next-generation sequencing (NGS), growing numbers of *CTCF* variants are being identified, and multiple genotype-phenotype databases cataloging such variants are emerging.

**Methods:**

In this study, we aimed to expand the genotypic spectrum of CRD, by cataloging NDD phenotypes associated with reported *CTCF* variants. Here, we systematically reviewed all known *CTCF* variants reported in case studies and large-scale exome sequencing cohorts. We also conducted a meta-analysis using public variant data from genotype-phenotype databases to identify additional *CTCF* variants, which we then curated and annotated.

**Results:**

From this combined approach, we report an additional 86 *CTCF* variants associated with NDD phenotypes that have not yet been described in the literature. Furthermore, we describe and explain inconsistencies in the quality of reported variants, which impairs the reuse of data for research of NDDs and other pathologies.

**Discussion:**

From this integrated analysis, we provide a comprehensive and annotated catalog of all currently known *CTCF* mutations associated with NDD phenotypes, to aid diagnostic applications, as well as translational and basic research.

## 1. Introduction

CCCTC-binding factor (*CTCF*) is a DNA-binding protein, equipped with 11 zinc fingers (ZFs) which facilitate its binding to thousands of sites across the genome (Lobanenkov et al., 1990; Splinter et al., 2006; Kim et al., 2007; Wendt et al., 2008; Pugacheva et al., 2015; Lobanenkov and Zentner, 2018). It is a universal regulator of 3D genome organization via the formation of chromatin loops and is a key transcriptional regulator (*CTCF* function has been extensively reviewed elsewhere) (Ohlsson et al., 2001; Klenova et al., 2002; Phillips and Corces, 2009). *CTCF* is ubiquitously expressed and highly conserved from *Drosophila* to humans, highlighting the importance of its correct structure and function within cells (Filippova et al., 1996; Moon et al., 2005).

Exome and whole-genome sequencing across thousands of human genomes has identified *CTCF* as a mutationally constrained gene, meaning that sequence variants are not well tolerated in the germline (Lek et al., 2016). *CTCF* variants are frequently identified in cancer; and *CTCF* haploinsufficiency is a known mechanism of tumorigenesis, highlighting *CTCF* as a tumor suppressor gene (Filippova et al., 1998; Rasko et al., 2001; Davoli et al., 2013; Kemp et al., 2014). As a result, large efforts have been made to elucidate the effects of *CTCF* depletion and mutations on genome architecture and gene expression, in a variety of model systems. Homozygous deletion of *CTCF* in mice results in early embryonic lethality, demonstrating the essential requirement of *CTCF* for viability (Wan et al., 2008; Moore et al., 2012). Hemizygous *CTCF* mice however, are viable and fertile, yet are predisposed to both spontaneous and induced tumor incidence, with global DNA methylation changes and deregulated gene expression patterns across tissues (Kemp et al., 2014; Alharbi et al., 2021). Depletion of *CTCF* in mammalian cell lines using the auxin-inducible degron system results in loss of chromatin looping and limited effects on gene transcription (Nora et al., 2017; Hyle et al., 2023). These studies highlight the necessity of correct *CTCF* gene dosage during development and throughout lifespan. Other studies conducted in cancer cell models have focused on the functional impact of *CTCF* mutations that disrupt the central ZF DNA-binding domain. Mutation of key residues to destroy the function of each zinc finger resulted in decreased DNA binding and *CTCF* residence time at binding sites (Nakahashi et al., 2013). Furthermore, several *in vitro* and *in silico* studies have also shown that specific cancer-associated mutations within *CTCF*, results in variable changes to cell growth, partial or complete loss of DNA binding in a site-specific manner, a reduction in chromatin residence time, loss of chromatin structure and aberrant transcription (Filippova et al., 2002; Bailey et al., 2021; Soochit et al., 2021). These studies also demonstrate the necessity of conserved *CTCF* structure and the range of genomic dysfunction that can result from mutation or loss of *CTCF*.

In 2013, Gregor et al. identified the first pathogenic *CTCF* variants in individuals diagnosed with neurodevelopmental disorder (NDD) phenotypes (Gregor et al., 2013). NDDs are a broad and heterogeneous group of conditions that are characterized by impairment of social, academic, personal or occupational functioning. Such conditions can include intellectual disorders (e.g., global developmental delay, intellectual disability), communication disorders, autism spectrum disorder, attention deficit hyperactivity disorder (ADHD), motor disorders and tic disorders (Wills, 2014). NDDs are heavily characterized by their neurological deficits, however they often present as syndromes affecting multiple systems in the body which lead to other notable phenotypes; including recurrent infections, congenital heart defects, urogenital and musculoskeletal anomalies, growth delay and craniofacial anomalies (Valverde de Morales et al., 2022). To date, 76 *CTCF* variants have been described in over 100 individuals that present with variable NDD phenotypes (Iossifov et al., 2014; Deciphering Developmental Disorders Study, 2015; Bastaki et al., 2017; Willsey et al., 2017; Chen et al., 2019; Konrad et al., 2019; Squeo et al., 2020; Wang et al., 2020; Hiraide et al., 2021; Valverde de Morales et al., 2022). NDDs caused by monoallelic pathogenic *CTCF* variants are now referred to as *CTCF*-related disorder (CRD) (ORPHA:363611).

Conditional knockout of *CTCF* in mouse neurons at various stages of development has produced phenotypes including disorganized brain development, increased neuronal apoptosis, behavioral and learning deficits, and premature death (Hirayama et al., 2012; Watson et al., 2014; Sams et al., 2016; Davis et al., 2022). Together, these studies highlight the central role that *CTCF* plays in maintaining correct 3D genome structure and gene expression, which are essential for proper neurodevelopment. These studies shed light on the pathogenic mechanism resulting from *CTCF* haploinsufficiency, however to date, no studies have yet explored the role of specific *CTCF* mutations found in NDD, in a neuronal model.

Due to the increasing use of exome sequencing in the clinic and in large-scale exome sequencing research projects in NDD cohorts, ever growing numbers of novel pathogenic variants continue to be identified and reported to genotype-phenotype data repositories worldwide (Srivastava et al., 2019). To the best of our knowledge, analysis of pathogenic *CTCF* variants implicated in NDD, utilizing public data, has not yet been conducted. In this study, our aim was to expand the current understanding of *CTCF* mutations that are associated with neurodevelopmental phenotypes. First, we performed a systematic review to identify all currently published cases of CRD. Second, we performed a meta-analysis on all *CTCF* variants submitted to genetic variant repositories, and identified those reported with NDD phenotypes. Herein, we provide an extensive catalog of *CTCF* mutations associated with NDD phenotypes, that have not yet been previously described in the literature.

## 2. Methods

### 2.1. Systematic review

A systematic review was conducted to identify published articles reporting *CTCF* variants associated with NDD phenotypes. Searches were conducted by two investigators (EP and LF), according to the Preferred Reporting Items for Systematic Reviews and Meta-analyses (PRISMA) guidelines (Page et al., 2021). Multiple searches were carried out in the PubMed database (https://pubmed.ncbi.nlm.nih.gov/) until 01 January 2023. No date restrictions were placed on the search. The search terms, inclusion and exclusion criteria used to select relevant studies are given in Table 1. Bibliographies of selected studies were also screened for relevant articles. This study did not require ethical board approval or written informed consent by the patients according to the study design (systematic review and data integration/meta-analysis).

**Table 1:** Search terms, inclusion, and exclusion criteria for systematic review.

**Category**	**Details**
Search terms	((*CTCF*) OR (CCCTC-binding factor) OR (next-generation sequencing)) AND ((mutation) OR (variant) OR (copy number) OR (deletion) OR (duplication)) AND ((neurodevelopment) OR (autism) OR (intellectual disability) OR (mental retardation))
Inclusion criteria for publications	Publication is written in English Publication is in peer-reviewed journal Study presents findings in human subject Study describes NDD conditions/phenotypes Study validates genetic cause of NDD with sanger sequencing/exome sequencing Study presents original data
Exclusion criteria for publications	Publication not in English Study describes mutation in non-human species (e.g., mouse model) Study topic not related to NDD (e.g., cancer) *CTCF* mutation classified as a *CTCF* binding site, not mutation of coding sequence

### 2.2. Data retrieval

We aggregated genetic variant data including copy number variants and sequence nucleotide variants from several sources; ranging from genotype-phenotype databases, published large-scale exome sequencing cohorts and case studies. We identified 11 genotype-phenotype databases for inclusion in this analysis. These were selected based on (1) data being publicly accessible and available for download, (2) *CTCF* variants were listed, (3) sufficient information including genomic coordinates and description of the variant being provided, and (4) reported associated phenotypes relevant to NDDs according to the DSM5 (Regier et al., 2013). We downloaded all *CTCF* variants alongside all available information from each of the following databases: ClinVar (Landrum et al., 2018) (https://www.ncbi.nlm.nih.gov/clinvar/), DECIPHER (Bragin et al., 2014) (https://www.deciphergenomics.org/), AutDB (Pereanu et al., 2018) (http://autism.mindspec.org/autdb/Welcome.do), Developmental Brain Disorder Gene Database (Mirzaa et al., 2014) (https://dbd.geisingeradmi.org/), Denovo-DB (https://denovo-db.gs.washington.edu/denovo-db/), DisGeNET (https://www.disgenet.org/search), EGIdb (Epilepsy Genetics, 2019) (http://egidb.org/), Gene4denovo (Zhao et al., 2020) (http://www.genemed.tech/gene4denovo/), LOVD (Fokkema et al., 2021) (https://www.lovd.nl/), SFARI (Arpi and Simpson, 2022) (https://gene.sfari.org/) and VariCarta (Belmadani et al., 2019) (https://varicarta.msl.ubc.ca/index). The final data search was performed across all databases on 02 February 2023. A brief description of each database is provided in Table 2. The following variables (when available) were extracted whether presented as text, figures, tables or Supplementary data; genomic coordinates (GRCh37/GRCh38), variant type (copy number variant/sequence variant), method of discovery (e.g. sequencing/array), inheritance (de novo/inherited), variant consequence (gain, loss, frameshift, nonsynonymous, synonymous), DNA sequence change, amino acid change and associated conditions/phenotypes. Any discrepancies in data extraction were discussed (by EP and LMF) before compiling the data into a single csv file for further data processing. Analysis of *CTCF* SNPs in the general population was performed using data from GnomAD (Karczewski et al., 2020) (https://gnomad.broadinstitute.org/), version 2.1.1 (last accessed: 04/23/2023).

**Table 2:** Genotype-phenotype databases used to download *CTCF* variant data.

**Source**	**Description**	**Version/release/last updated**	**Variant entries**	**Citation**
AutDB	A reference for all known genes associated with ASD	September, 2022	25,393	Pereanu et al., 2018
EGIdb	Genetic variants from patients sequenced for epilepsy	v1 / October 03, 2017	NA	Epilepsy Genetics, 2019
DisGeNet	Collection of genes and variants associated with human diseases	v7.0 / June 2020	1,134,942	Piñero et al., 2017
Varicarta	Variants found in ASD and reported in peer-reviewed scientific literature	November 09, 2022	210,602	Belmadani et al., 2019
DECIPHER	Genomic variants of patients who have been evaluated in a clinic or are part of a research study	v11.17 / December 14, 2022	58,837	Bragin et al., 2014
LOVD	Database of genomic variants and phenotypes	v.3.0 / June 15, 2021	844,462	Fokkema et al., 2021
SFARI	Comprehensive reference of all known human genes associated with ASD from peer-reviewed journals	February 23, 2022	3,803	Arpi and Simpson, 2022
Denovo DB	De-novo variants identified in human genome	v.1.6.1 / August 19, 2018	NA	Turner et al., 2017
Gene4denovo	De novo mutations in humans	July 08, 2022	741,866	Zhao et al., 2020
ClinVar	Genetic variants and associated phenotypes	January 05, 2023	1,660,725	Landrum et al., 2018
DBD	Developmental Brain Disorder Gene Database	September 01, 2022	11,276	Mirzaa et al., 2014

### 2.3. Data curation

Data was formatted differently depending on its source. Thus, all data was standardized and compiled into a dataset containing all variants. The compiled dataset was processed to ensure the variants it contained were interoperable and could be analyzed as a single dataset, regardless of its source (Ehrhart et al., 2021). All coordinates were converted to GRCh37 using the LiftOver (Kuhn et al., 2012) tool provided by UCSC (http://genome.ucsc.edu/cgi-bin/hgLiftOver). Manual annotation was performed for any variant that did not provide suitable genomic coordinates for conversion. Any ambiguous variants were excluded from analysis. All sequence nucleotide variants were mapped against the canonical *CTCF* transcript (NCBI Reference Sequence: NM_006565.4). All variant nomenclature was standardized according to HGVS using the Mutalyzer3 tool (https://mutalyzer.nl/) (Wildeman et al., 2008).

### 2.4. Variant annotation

All data processing, organization and visualization was conducted using R (version 4.2.2) and R studio. Downloaded R packages included tidyverse and ggplot2. Genomic descriptions were added to each variant based on its location across the *CTCF* gene sequence (i.e., exonic, intronic or UTR) using coordinates provided for transcript ENST00000264010.4 in Ensembl (https://grch37.ensembl.org/) (last accessed: 02 February 2023) (Cunningham et al., 2022). Annotations were also added describing which protein coding domain each variant affected (i.e., zinc-finger domain, N term or C term). The pathogenicity of *CTCF* variants was assigned according to the AGMC guidelines (Richards et al., 2015). Exonic variants were also scored using PolyPhen to predict the impact of protein coding substitutions (Adzhubei et al., 2013).

### 2.5. Phenotype analysis

The diagnosis of NDD currently follows the guidelines set forth by the DSM-5 (Regier et al., 2013). To characterize *CTCF* variants associated with NDD, phenotypic information was manually reviewed for inclusion of terminology that either categorically stated a diagnosis of CRD - or a description of NDD more broadly. As CRD is a relatively new term (Valverde de Morales et al., 2022), and medical terminology for rare disease is frequently updated, a diagnosis of CRD or NDD was counted if previous terminology was used; including “Mental retardation, autosomal dominant 21” or “MRD21 (Intellectual disability-feeding difficulties-developmental delay-microcephaly syndrome)”. When a specific diagnosis of CRD was not provided, additional diagnostic terminology that is characteristic of NDD was included. An overview of this terminology is given in Table 3. Furthermore, the clinical features (as listed in the human phenotype ontology) describing CRD were also used when reviewing phenotypic information, to ascertain if the phenotype was consistent with CRD/NDD. These are provided in Supplementary material 1.

**Table 3:** Reported conditions/phenotypes screened for in genetic variant datasets.

**Condition**	**Classification**	**Description**	**Identifiers**
*CTCF* related neurodevelopmental disorder (CRD)	Mental/behavioral dysfunction	Rare, genetic, neurodevelopmental disorder Caused by heterozygous pathogenic *CTCF* variants See clinical features	MedGen UID: 816016. Concept ID: 3809686
Intellectual disability (ID)	Mental/behavioral dysfunction	Subnormal intellectual functioning Originates during developmental period IQ score below 70	MedGen UID: 811461. Concept ID: 3714756
Developmental disorder (DD)	Mental/behavioral dysfunction	Diagnosed in childhood Physical/mental impairment Affects reaching age related developmental milestones	MedGen UID: 3367. Concept ID: 0008073
Inborn genetic diseases (IGD)	Disease/syndrome	Caused by genetic mutations present during development Mutations may be inherited/de novo	MedGen UID: 181981. Concept ID: 0950123
Epilepsy (EP)	Disease/syndrome	Characterized by recurrent seizures	MedGen UID: 4506. Concept ID: 0014544
Autism spectrum disorder (ASD)	Mental/behavioral dysfunction	Limited/absent verbal communication Lack of reciprocal social interaction or responsiveness Restricted, stereotypic, and ritualized patterns of interests and behavior	MedGen UID: 307153 Concept ID: 1510586
Congenital nervous system disorder (Other)	Congenital abnormality	Abnormality of the nervous system See clinical features (see Supplementary Table 1)	MedGen UID: 105425 Concept ID: 0497552

## 3. Results

### 3.1. Generation of *CTCF* variant dataset

#### 3.1.1. Systematic review did not yield any new *CTCF* variants

**Figure 1:**
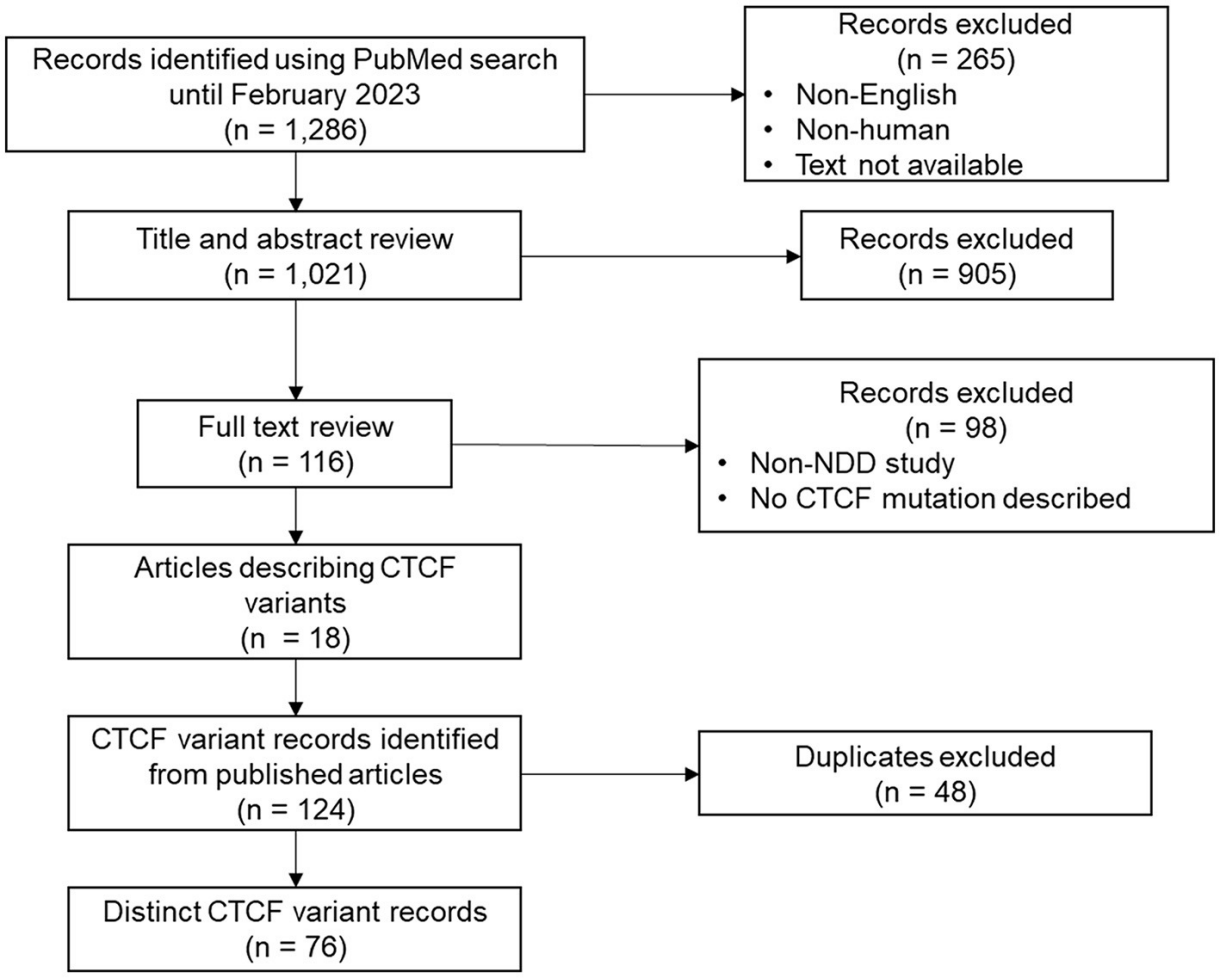
Overview of systematic review. Process of data search, identification, and filtering.

To provide a comprehensive catalog of *CTCF* variants associated with NDD phenotypes, a systematic review was first conducted to identify all *CTCF* variants discovered in probands with diagnosed NDDs. The literature search yielded 1,286 article records (Figure 1). After records were filtered for being written in English, presenting human findings and having the full text available, 1,021 results remained. Titles and abstracts were manually screened, leaving 116 records for full text review. Search results contained both case studies/series that highlighted *CTCF* variants found in specific probands, and large-scale next generation sequencing (NGS) studies that performed either whole-genome sequencing, or exome sequencing on cohorts with a presenting NDD phenotype. NGS studies did not categorically mention *CTCF* variants in either title, abstract or main text. Therefore, supplementary NGS data was further reviewed to identify *CTCF* variants. As expected, when a *CTCF* variant was reported in a large-scale NGS cohort, the phenotypic detail of the affected proband was minimal in contrast to highly descriptive *CTCF* variant case studies. In addition to extensively reviewing the articles obtained from the systematic review, citation lists were also screened to identify other potentially relevant studies that may have been missed. In total this systematic review identified that *CTCF* variants were reported 124 times from the 18 publications that were screened (Figure 1). After duplicates were removed, this corresponded to 76 distinct genetic *CTCF* variants associated with an NDD phenotype that had already been previously summarized (Valverde de Morales et al., 2022).

#### 3.1.2. Data aggregation revealed many *CTCF* variant entries in genotype-phenotype databases

**Figure 2:**
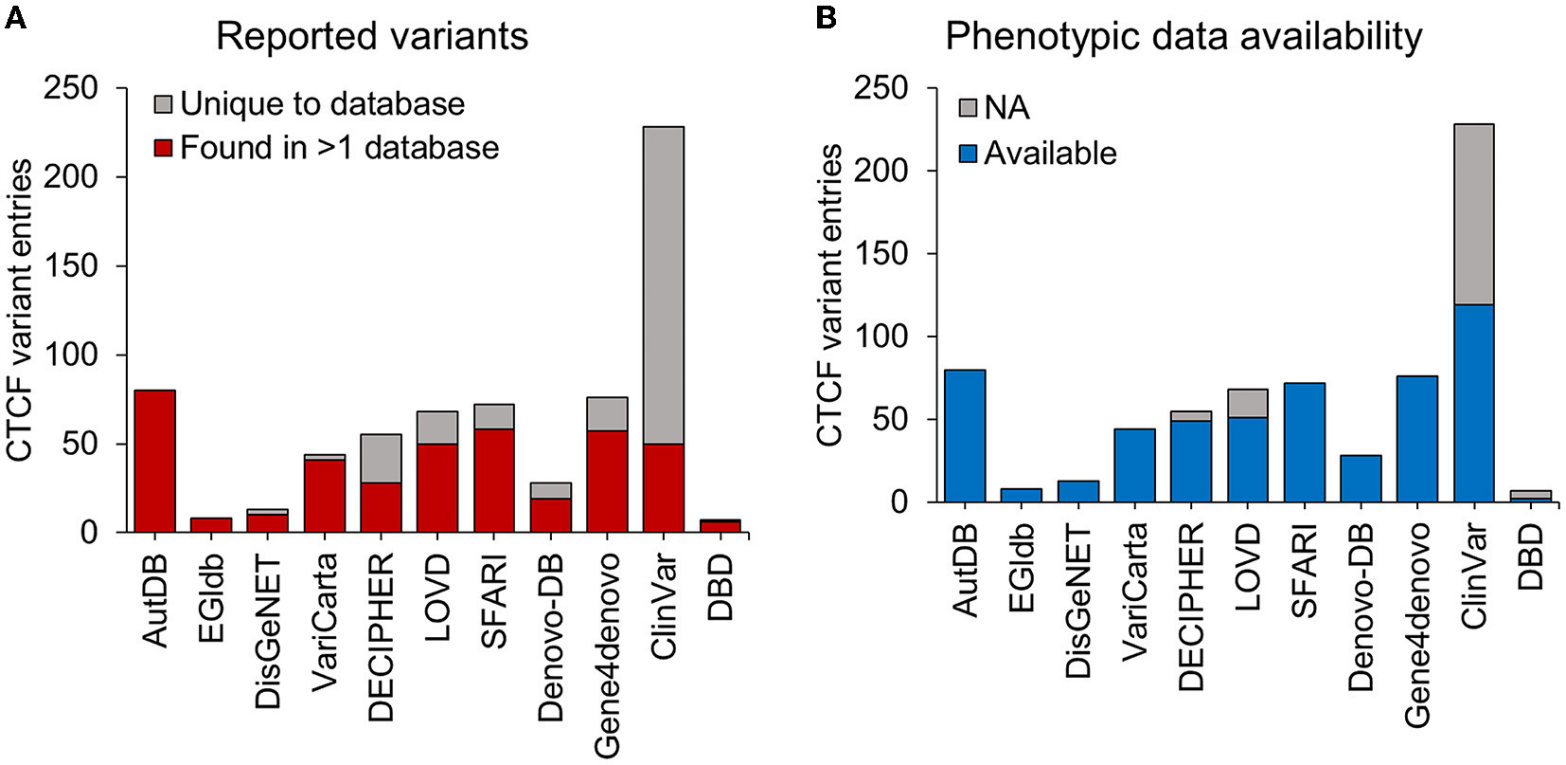
*CTCF* variants reported in genotype-phenotype databases. Stacked bars represent the total number of *CTCF* variant entries (y-axis) retrieved from different genotype-phenotype databases (x-axis). **(A)** The total number of *CTCF* variant entries that were uniquely reported within a database (gray) and variants found in at least one other database (red). **(B)** The total number of *CTCF* variants entries reported with available phenotypic data (blue) and those without any phenotypic data (gray). NA, not available.

In addition to the variants identified from the systematic literature review, we aimed to identify other *CTCF* variants associated with NDDs that had not been reported in the literature. We downloaded data describing *CTCF* variants from 11 databases reporting genotype-phenotype associations (Table 4). Some databases contained variants associated with a specific disorder. For example, SFARI (Arpi and Simpson, 2022) only contained variants associated with autism, whereas other databases contained variants from a broad range of phenotypes [e.g., ClinVar (Landrum et al., 2018)]. From the data retrieval, we generated a comprehensive dataset that contained 679 *CTCF* variant records in total (Table 4). The greatest number of *CTCF* variant entries were reported by ClinVar (228, 33%), AutDB (80, 11.9%), Gene4denovo (76, 11.2%), SFARI (72, 10.7%) and LOVD (68, 10.1%) (Table 4, Figure 2). Of note, AutDB and EGIdb did not contain any unique *CTCF* variant entries. Phenotypic data was available for 80% of *CTCF* variant records, however this varied greatly between databases (Figure 2B). For example, ClinVar contained the greatest number of unique *CTCF* entries (Figure 2A), but phenotypic data was unavailable for approximately half of these (48%), whereas 100% of entries had phenotypic information available in Gene4denovo (Figure 2B). The variants identified from the systematic literature review and data retrieval were compiled into a single dataset for further analysis.

**Table 4:** Summary of *CTCF* variants listed in each database.

**Database**	**Genotype-phenotype associations**	**All variant entries**		**Unique variant entries**		
		**Count**	**Proportion of all entries**	**Count**	**Exclusive to database**	**Proportion of all entries**
ClinVar	Mixed	228	33.8%	178	78.1%	26.4%
AutDB	ASD	80	11.9%	0	0.00%	0.00%
Gene4denovo	Mixed	76	11.2%	19	25.0%	2.8%
SFARI	ASD	72	10.7%	14	19.4%	2.1%
LOVD	Mixed	68	10.1%	18	26.5%	2.7%
DECIPHER	Mixed	55	8%	27	49.1%	4.0%
VariCarta	ASD	44	6.5%	3	6.8%	0.04%
Denovo-DB	Mixed	28	4.2%	9	32.1%	1.3%
DisGeNET	Mixed	13	1.9%	3	23.1%	0.04%
EGldb	Epilepsy	8	1.2%	0	0.00%	0.00%
DBD	NDD	7	1.0%	1	14.3%	0.2%
Total		679	100%	272	n/a	39.6%

### 3.2. *CTCF* sequence nucleotide variants associated with NDD phenotypes

#### 3.2.1. Noncoding *CTCF* SNVs

**Figure 3:**
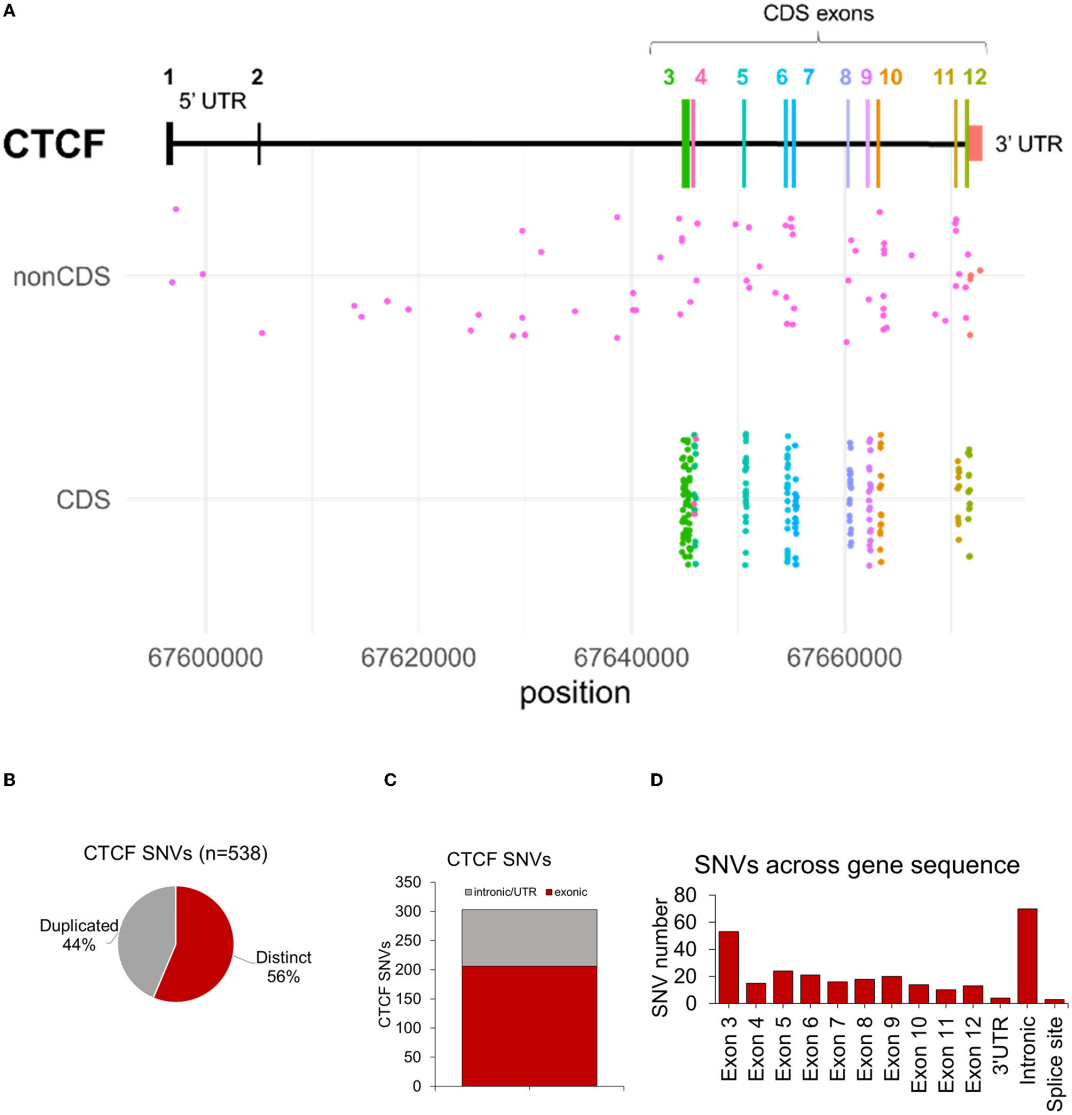
Summary of *CTCF* exonic SNVs associated with NDD phenotypes. **(A)** Plot showing structure of *CTCF* gene. X axis indicates *CTCF* variants associated with NDD phenotypes in either non-coding sequence (nonCDS) and coding sequence/exonic (CDS) regions. Y axis indicates the chromosome position of each variant. **(B)** Number of duplicated and distinct *CTCF* sequence nucleotide variants (SNVs) identified from data retrieval and systematic literature review. **(C)** Number of variants in intronic/UTRs versus exonic sequences. **(D)** Number of distinct CNVs across the *CTCF* gene sequence after duplicates were removed.

In the human genome (GRCh37) the *CTCF* canonical gene sequence (RefSeq: NM_006565.4, Ensemble: ENST00000264010.4) is encoded at chr16q22.1 (chr16:67,596,310-67,673,088), spanning 76,779 bp across 12 exons (including UTRs in exons 1, 2 and 12) (Figure 3A). The protein coding sequence for *CTCF* (chr16:67,644,736–67,671,775) encodes 27,040 bp in total, across 10 coding exons (exons 3-12). Sequence nucleotide variants (SNVs) including base substitutions, small deletions/duplications and insertions were analyzed first. 538 SNV entries were identified, of which 44% were duplicate variants (Figure 3B). After removing duplicates, 311 distinct SNVs were identified across the entire *CTCF* gene sequence (Figure 3C). In total, 86 SNVs were in noncoding sequences (introns and 3′ UTR) (Figure 3C). No variants were identified in the 5′ UTR (Figure 3D). In total, 31 noncoding SNVs (ncSNVs) were associated with an NDD phenotype. 24 ncSNVs were reported in association with ASD, 6 ncSNVs in cases of CRD and 1 ncSV was reported in a case of abnormality of the nervous system (Supplementary material 2). 46 ncSNVs did not report any associated phenotype and 9 ncSNVs were detected in controls (i.e., participants included in sequencing cohorts without NDD-phenotype). Whilst a description of ncSNVs is provided here, it is difficult to predict their pathogenic mechanism, therefore we have not analyzed them further or included them as part of the NDD genotypic spectrum.

#### 3.2.2. Exonic *CTCF* SNVs

As pathogenic *CTCF* variants previously associated with CRD have been shown to affect the protein coding exons, this remained the focus of our analysis. After filtering out variants which affected protein coding exons and removing duplicate entries, 225 *CTCF* exonic variants remained. The main aim of this study was to broaden the genotypic spectrum of NDD related to *CTCF*, therefore all exonic variants identified from the data retrieval were reviewed for phenotypic information and manually annotated within the dataset. Those that were categorized as being associated with an NDD phenotype were based on the criteria listed in Table 3, Supplementary Table 1. Qualifying NDD phenotypes included a clinical diagnosis of CRD, autism spectrum disorder (ASD), developmental disorder (DD), epilepsy (EP), intellectual disability (ID), inborn genetic disease (IGD) and abnormality of the nervous system (ANS). In total, 149 out of 225 (66%) exonic *CTCF* variants were found to be reported in association with an NDD phenotype. Seven out of 225 (3%) exonic *CTCF* variants from the data retrieval were reported in association with either a non-NDD phenotype or a phenotype that did not qualify as NDD due to limiting information. These phenotypes included mammary neoplasms/breast cancer, acute megakaryoblastic leukemia in down syndrome and congenital diaphragmatic hernia. These variants were excluded from further analysis. 70 out of 225 (31%) exonic *CTCF* variants did not report any phenotypic data, and thus were also excluded from further analysis.

#### 3.2.3. NDD phenotypes associated with exonic *CTCF* variants

**Figure 4:**
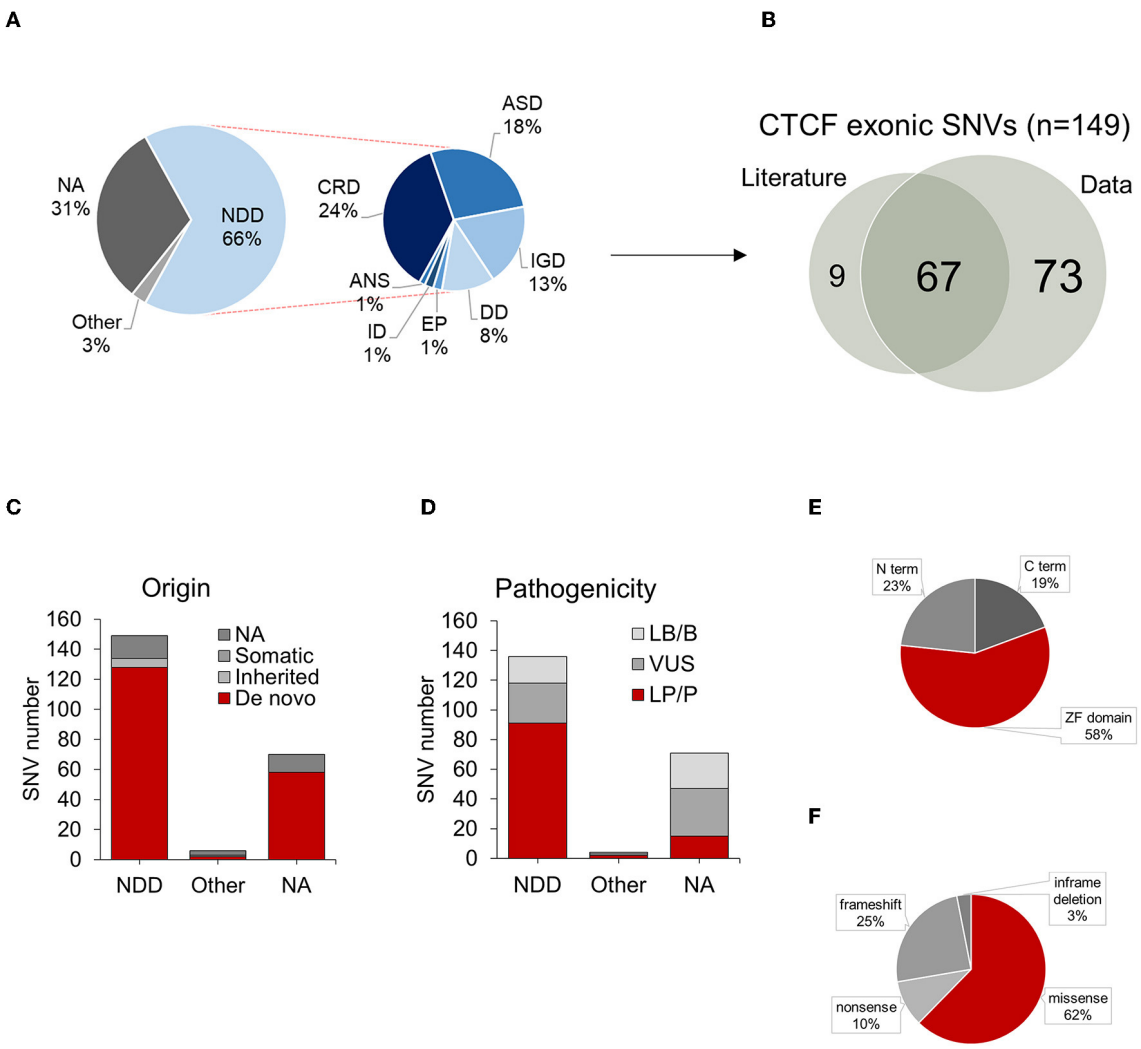
Summary of *CTCF* exonic SNVs associated with NDD phenotypes. **(A)** Proportion of NDD phenotypes associated with *CTCF* exonic SNVs. **(B)** Overlap of exonic *CTCF* variants identified in literature and data retrieval process. **(C)** Origin of *CTCF* exonic SNVs categorized by associated phenotypes. **(D)** Pathogenicity of *CTCF* exonic SNVs. Summary of mutation types for SNVs categorized by associated phenotypes. **(E)** Distribution of exonic SNVs across protein domains, **(F)** Summary of mutation types for SNVs reported in association with NDD phenotypes. NDD, neurodevelopmental disorder; SNVs, single nucleotide variant;NA, not available.

The most common phenotype reported in association with exonic *CTCF* variants was CRD (24%), followed by ASD (18%), IGD (13%), DD (8%), EP (1%), ID (1%) and ANS (1%) (Figure 4A). A full overview of reported phenotypes, with references to original sources for additional information is provided in Supplementary Table 2. Exonic *CTCF* variants retrieved from the data integration analysis were cross referenced with those previously reported in the literature. 73 out of 149 (49%) exonic *CTCF* variants associated with NDD phenotypes were found exclusively from the data aggregation. As previously mentioned, 76 *CTCF* variants were identified from the systematic review of the published literature, which overlapped with 67 (45%) of the variants found in our data aggregation (Figure 4B). 9 (6%) variants were exclusively reported in the literature and not documented in any database included in this study (Figure 4B). We also plotted each mutation type based on the classification of NDD phenotype (Supplementary Figure 1) however we did not observe any phenotype-specific clustering.

#### 3.2.4. Origin of exonic *CTCF* variants associated with NDD phenotypes

We also explored the mode of inheritance for each variant based on the availability of trio-exome sequencing performed on the proband and both biological mother and father. 128 out of 149 (85%) exonic *CTCF* variants were confirmed to be of germline *de novo* origin, 6 out of 149 (4%) were inherited and 15 out of 140 (11%) were of unconfirmed origin, due to a lack of trio-exome sequencing being performed (Figure 4C). As described in previous studies (Konrad et al., 2019; Valverde de Morales et al., 2022), the majority of NDD associated *CTCF* variants are *de novo* germline variants, however a small number were confirmed to be inherited. Further studies are required to elucidate the penetrance of CRD.

#### 3.2.5. Pathogenicity of exonic *CTCF* variants associated with NDD phenotypes

When available, we reviewed the provided pathogenicity score for each variant however some of the entries were reported as early as 2011, prior to the first described case study of CRD–therefore all variants were manually reviewed and reclassified according to the current AMGC guidelines, with further insights provided by recently available experimental data exploring the role of *CTCF* mutations in cell assays and other experimental models. 91 (61%) of exonic variants were classed as pathogenic (P) or likely pathogenic (LP), 27 (18%) were classed as a variant of unknown significance (VUS), and 18 (12%) were classed as benign (B) or likely benign (LB) (Figure 4D). Upon further inspection, we identified that many LB/B variants that were reported in association with an NDD phenotype were actually synonymous mutations (e.g., p.Val6=). Due to the unlikely nature of a synonymous mutation in *CTCF* being pathogenic, all synonymous variants were removed from the analysis. Some variants originally classed as LB/B were missense mutations; e.g., an inherited p.Asp46Asn affecting the N terminus, a de novo p.Arg415Gln affecting ZF6 and two de novo p.Pro643Ser and p.Ala697Thr both affecting the C terminus. These remained in the dataset as they were reported in association with NDD phenotypes however they were reclassified as a VUS (see Supplementary Table 2).

#### 3.2.6. Pathogenic *CTCF* variants cluster across zinc finger domain

**Figure 5:**
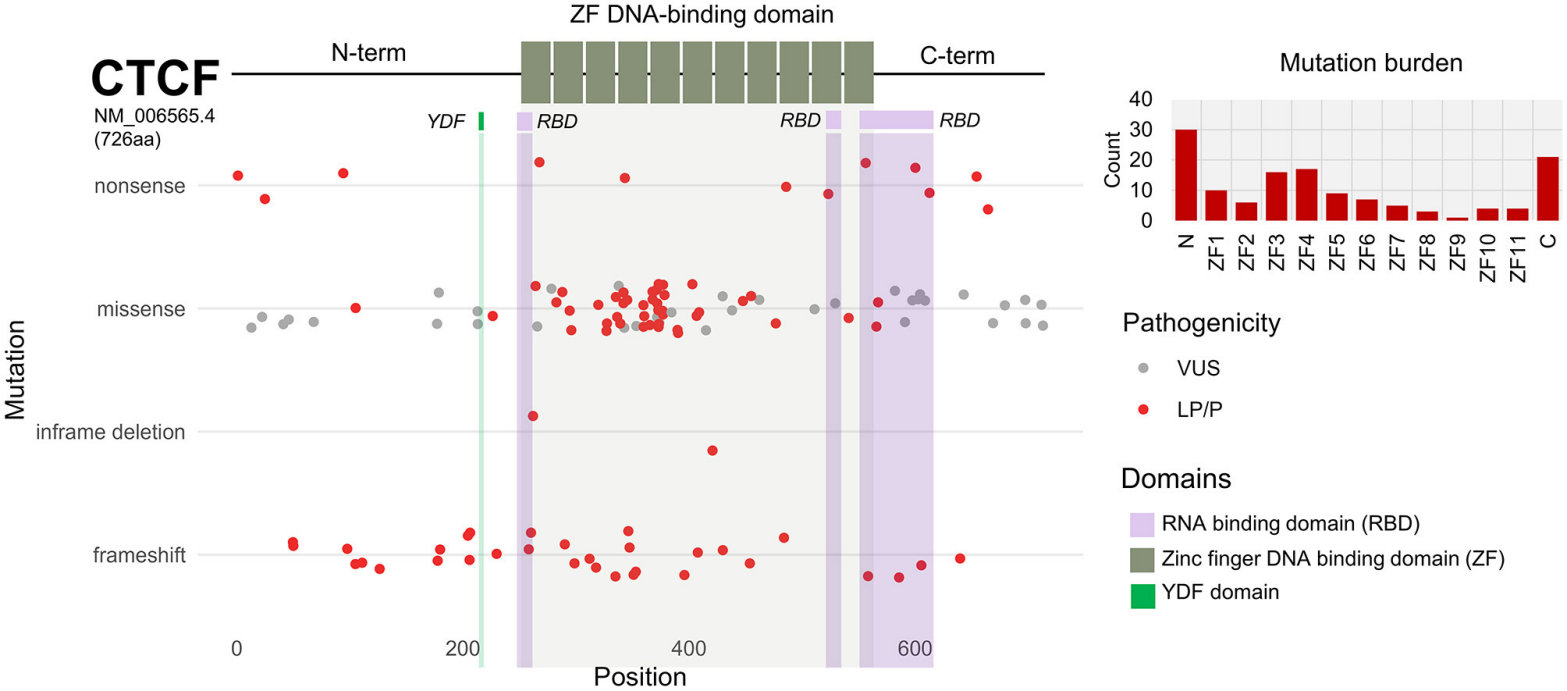
*CTCF* exonic sequence nucleotide variants (SNVs) associated with NDD phenotypes. Schematic of *CTCF* protein structure (NM_006565.4) encoding 726 amino acids. N and C termini are depicted by black line. Central DNA-binding zinc-fingers (ZFs) 1 to 11 are shown by gray boxes. Mutational burden of N terminus, ZFs and C terminus is shown as bar chart. Scatterplot shows exonic SNVs are plotted according to corresponding amino acid position (x-axis). SNVs are categorized based on mutational consequence (y-axis). Clinical significance and pathogenicity of each SNV is indicated by color; VUS = gray, LP/P = red.

In total, there were 134 nonsynonymous coding variants that were included in this analysis, which corresponded to 127 protein changes. The majority of these variants were located across the zinc finger domain (Figure 4E). This is because in some cases, different genetic variants resulted in the same amino acid substitution. 62% of nonsynonymous mutations were missense (Figure 4F). 32 out of 134 mutations resulted in a frameshift. For example, a confirmed *de novo* c.604dupA variant resulted in p.Thr204Asnfs^*^25 which causes a frameshift mutation in the N terminus resulting in the loss of function of one of the *CTCF* alleles. 4 variants resulted in an in-frame deletion and 13 variants resulted in the gain of an early termination (TAA/TAG/TGA) signal resulting in a nonsense mutation. To further investigate the functional consequence of *CTCF* variants associated with NDD, we plotted each nonsynonymous exonic *CTCF* variant across the protein sequence based on its mutational consequence and pathogenicity/clinical significance (Figure 5). We observed an enrichment of pathogenic missense mutations across the ZF domain with a particular enrichment in ZF3 and ZF4 (Figure 5). Interestingly, these are the same ZFs that have elevated levels of mutations in cancer (Bailey et al., 2021). ZF 4 to 7 bind to the core *CTCF* motif, and previous attempts to obtain cell lines with mutant ZF 2 to 7 were unsuccessful, demonstrating the essential nature of these key binding fingers for cell viability (Nakahashi et al., 2013; Soochit et al., 2021). Previous studies identified pathogenic mutations in all ZFs except ZF8 and ZF9 (Valverde de Morales et al., 2022). Here, we provide novel examples of mutations in ZF8 and ZF9 being associated with NDD phenotypes. For example, c.1456C>T p.Gln486Ter is a pathogenic germline mutation reported in a case of IGD and c.1430A>C p.His477Pro is reported in a case of ASD. Deletion of ZF8 has been shown to reduce chromatin residence time, chromatin looping and alter gene expression (Soochit et al., 2021). The effect of these specific mutations should be investigated functionally.

**Figure 6:**
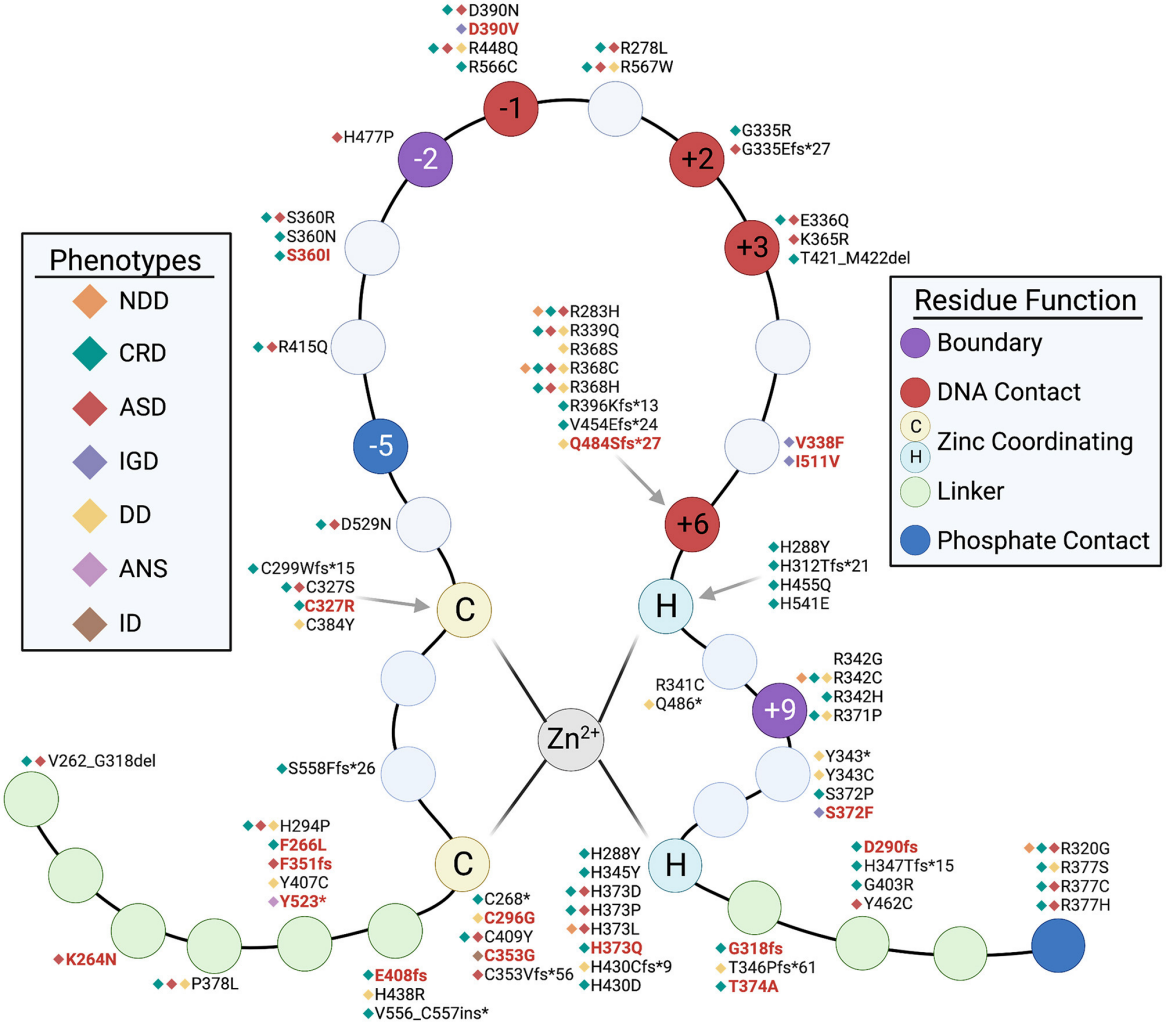
Structure of the *CTCF* zinc-finger, indicating key residues affected by NDD-associated mutations. C denotes Cysteine residue, H denotes Histidine residue. Mutations associated with NDD phenotypes are annotated. Red text indicates new mutation identified in this study. Black text indicates it has been reported previously. Keys refer to specific NDD phenotype reported in association with mutation and function of residues.

We investigated the mutations across the ZF region in further detail to see which specific residues were affected (Figure 6). Consistent with findings published by Valverde et al., additional missense mutations identified by this analysis also targetted the ZF domain and affect key residues that are critical for ZF function. Many mutations were found in all key Cysteine and Histidine zinc coordinating residues (e.g., C353G, C271W, H541E and H345Y). Mutation of zinc coordinating residues across all 11 ZF has shown to reduce *CTCF* binding and residency time at binding sites, demonstrating how zinc binding residues in all zinc fingers are critical for the proper functioning of *CTCF*, and without it, *CTCF* loses its ability to bind its cognate recognition sequences (Ohlsson et al., 2001; Nakahashi et al., 2013; Soochit et al., 2021). Other mutations affect residues at ZF positions −1, +2, +3 and +6 that are essential for direct contact with DNA (Filippova et al., 2002; Bailey et al., 2021). Aside from the central ZF DNA binding domain there are mutations in the N and C termini which contain additional functional domains. One mutation that has been previously reported (c.677A>G p.Tyr226Cys) affect the YDF domain in the N term at position 226–228. Functional studies have shown that while a mutated N-terminal YDF domain does not affect *CTCF* binding across the genome, it impairs the ability of *CTCF* to pause and retain cohesin binding associated with the loss of chromatin looping (Li et al., 2020; Pugacheva et al., 2020). This highlights how mutations outside of the ZF DNA binding domain can also be pathogenic via a different mechanism of action. Other data has shown that ZF1 (position 264–275) and ZF10 (position 536–544) contain RNA-binding domains (RBDs) which are important to maintain chromatin binding and the formation of chromatin loops (Saldaña-Meyer et al., 2019). A functional RBD also exists in the N terminus which affects the ability of *CTCF* self-interact (Saldaña-Meyer et al., 2014; Hansen et al., 2019). Whilst no major impact to genome organization was observed in RBD mutants, some gene expression differences have been observed. Mutations in the RNA binding domains of *CTCF* in NDD cases have been previously described elsewhere (Valverde de Morales et al., 2022) (e.g., c.804_805del p.Cys268Ter). However, new variants were also found in this study including c.798C>G p.Phe266Leu and c.792G>C p.Lys264Asn in the RBD located at ZF1. Interestingly, both affect the same RBD yet one is classed as LP and one is classed as VUS.

### 3.3. *CTCF* SNPs in the general population are most frequent in 3′ UTR

To better understand NDD-associated variants and their distribution across *CTCF*, we analyzed *CTCF* SNPs from the GnomAD database, which compiles variants from 125,748 exome sequences and 15,708 whole-genome sequences, representing the general human population (Karczewski et al., 2020). Whilst efforts are made to remove pediatric disease from this reference dataset, this is not 100% guaranteed (particularly when using data from biobanks). We identified *CTCF* variants present in 40,246 human genomes (32%) corresponding to 753 distinct variants in total. 99% were classified as rare (allele frequency <0.05), which was expected due to *CTCF* being highly conserved and mutationally constrained. Only 2 SNPs were identified as common (allele frequency >0.05). One SNP (rs6499137) was in the 3′UTR encoding c.^*^29T>G and the other SNP (rs143837268) encodes a synonymous p.Ser388Ser mutation (c.1164C>T) in zinc finger 5. This synonymous mutation was identified in our search as being reported in cases of epilepsy and inborn genetic disease but were both classified as benign (Supplementary Table 2). Further analysis of these 2 SNPs revealed population differences (Supplementary Figure 2A). The 3′ UTR variant is common in all populations except people of east Asian ancestry. Whereas the ZF5 variant is common to individuals with European (Finnish) ancestry only. Data was unavailable to explore the ethnicity of individuals with NDD associated *CTCF* variants, however this should be assessed in the future as more data becomes available.

Based on total allele counts, 3′ UTR variants were the most common, identified in nearly 30,000 genomes, followed by exonic synonymous variants, intronic, and then exonic missense variants (Supplementary Figure 2B). As expected, no frameshift variants were reported, consistent with the pathogenic haploinsufficiency model of NDD resulting from loss of *CTCF* (Hirayama et al., 2012; Watson et al., 2014; Sams et al., 2016; Davis et al., 2022). 29% of SNPs were located within exons. We plotted these variants across the protein structure of *CTCF* (Supplementary Figure 2C). We observed a consistent distribution of synonymous variants across the entire length of the protein however we observed a decreased enrichment of missense mutations across the zinc finger domain compared to the N and C terminus. This is the opposite of the trend we observed in NDD associated mutations, which showed an enrichment of missense mutations across the zinc finger domain. This is consistent with the mutational constraint of *CTCF*, particularly across its zinc finger domain which is essential to maintain its DNA binding function (Ohlsson et al., 2001; Filippova et al., 2002; Nakahashi et al., 2013; Hiraide et al., 2021; Soochit et al., 2021).

### 3.4. *CTCF* copy number variants associated with NDD phenotypes

**Figure 7:**
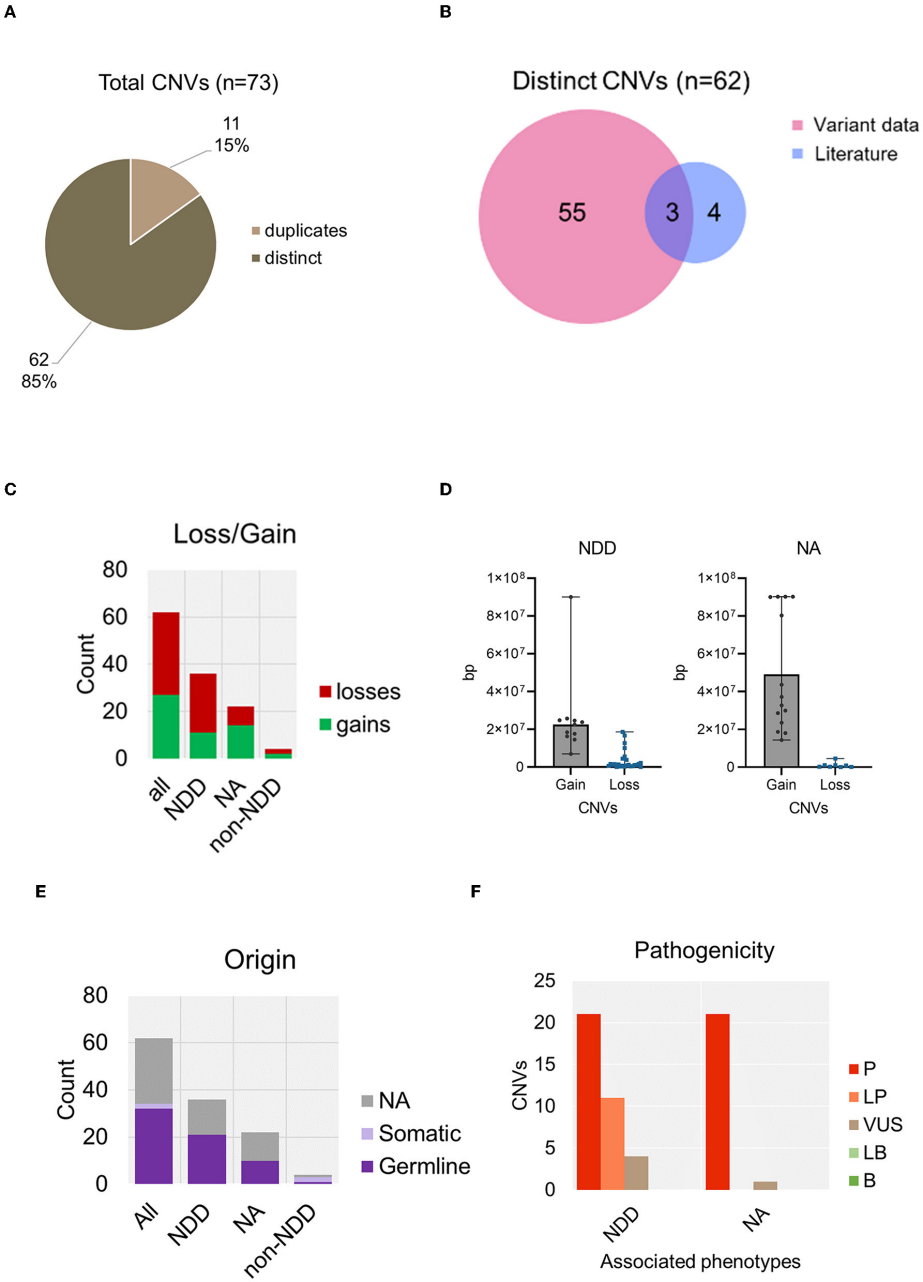
Summary of copy number variants containing *CTCF*, associated with NDD phenotypes. **(A)** Total number of copy number variants containing *CTCF* identified in analysis. **(B)** Number of distinct CNVs identified from data retrieval versus those already reported in the literature. **(C)** CNV loss and gains identified in association with NDD versus those that did not report a phenotype or a non-NDD phenotype. **(D)** Size analysis of *CTCF* CNVs associated with NDD compared to non-NDD phenotype. **(E)** Origin of CNV categorized by associated phenotype. **(F)** Pathogenicity of CNVs categorized by associated phenotype. NA, not available.

From our data integration and analysis of published CRD case studies, we identified a total number of 73 records describing copy number variants (CNVs). 11 CNVs (15%) were duplicates (Figure 7A). As no clinically identifying information was available, it could not be determined if these entries were duplicates from the same individual. Therefore, duplicates with the same genomic coordinates were removed. In total we report 62 distinct CNVs (Supplementary Table 3). 7 of these CNVs associated with CRD were previously reported in the literature (Gregor et al., 2013; Hori et al., 2017; Konrad et al., 2019; Valverde de Morales et al., 2022), 3 overlapped with our data and 4 were not reported in any genotype-phenotype database (Figure 7B). 27 CNVs were gains and 35 CNVs were losses (Figure 7C). As previously stated, CNV records were analyzed for reported NDD phenotypes. 36 CNVs were confirmed in cases of CRD or DD. Notably, the size ranges between gains and losses differed. CNV gains associated with NDD phenotypes were generally very large and ranged between 5 Mb to 90 Mb whereas losses ranged from a much smaller deletion size of 1.4 kb to a larger 44 Mb (Figure 7D). Of these, 21 CNVs were confirmed to be *de novo* (Figure 7E). Furthermore, 32 of these variants were classed as LP/P and 2 were VUS (Figure 7F). This data analysis reports an additional 29 CNVs that are associated with NDD phenotypes that were not previously reported in the literature. No translocations were described.

## 4. Discussion

### 4.1. Advantages of genotype-phenotype databases in profiling *CTCF* variants in NDD

In this comprehensive analysis, we searched for all *CTCF* variants associated with NDD phenotypes. Through a systematic review of the literature and data retrieval from genotype-phenotype databases, we report 163 distinct *CTCF* variants associated with NDD phenotypes. The most comprehensive case series to date by Valverde et al. reported 76 *CTCF* variants in 104 individuals diagnosed with CRD (Valverde de Morales et al., 2022). Our systematic literature did not identify any new variants that were not already included in the Valverde study. In contrast, our approach of assimilating variant data from genotype-phenotype databases resulted in the identification of many novel *CTCF* variants that were submitted from large-scale NGS studies that were missed during the systematic review (Krumm et al., 2015; Cappi et al., 2020; Kaplanis et al., 2020; Brunet et al., 2021; Zhou et al., 2022). Examples include a study by Kaplanis et al. (2020), who sequenced 31,058 parent–offspring trios of individuals with NDDs and reported the pathogenic *CTCF* variant c.1813delA p.Lys605Argfs^*^25 to the Gene4denovo database. Another example by Brunet et al. (2021), performed parent-offspring trio exome sequencing in 231 individuals with NDDs and reported c.958C>G p.Arg320Gly in an individual with ASD to the SFARI database. Additionally, this approach enabled us to review variants that were reported by diagnostic exome-sequencing service providers, like Gene xD (https://www.genedx.com/), who have submitted 83 records of *CTCF* variants to the ClinVar database since 2011. All variants with references to their source publication are provided in Supplementary Tables 2, 3 to serve as a resource for clinicians and researchers.

### 4.2. Limitations of genotype-phenotype databases in profiling *CTCF* variants in NDD

One limitation of reviewing variant data without access to patient data was the inability to distinguish between duplicate entries reported across several databases. 60% of *CTCF* variants found during the data retrieval were found in at least 2 different datasets. Duplicates were removed to provide a list of distinct variants and avoid redundancy, however this meant that we could not assess variant frequencies. The best description of recurrent *CTCF* variants in different NDD subjects has been provided by the Valverde study (Valverde de Morales et al., 2022). Furthermore, we observed that only 40% of *CTCF* variants were unique to a single database. This highlights a lack of consistency in reporting novel *CTCF* variants and a caveat in data sharing between available genomic resources. Chromosomal microarrays are usually the first-tier test for NDDs, yet the majority of CRD cases to date have been diagnosed through multigene panel or exome sequencing which detect a mutation in the protein coding sequence (Srivastava et al., 2019). As healthcare and diagnostics move toward NGS and a genotype first approach, efforts should be made to make genomic data FAIR (findable, accessible, interoperable and reusable) (Corpas et al., 2018). Improving consistency when reporting of genomic patient data can improve diagnostics in the future. Another limitation of this study was the lack of available phenotypic data, which varied between genotype-phenotype databases. For example, 48% of *CTCF* variants reported in ClinVar did not have any accompanying phenotypic data compared to Gene4Denovo that reported phenotypic information for 100% of *CTCF* variants listed (see Figure 2B). Our strategy during this analysis was to profile those variants which could be associated with NDD phenotypes according to human phenotype ontology terms, therefore many variants without any associated phenotypic data were excluded from the study. Therefore, it is likely that we have excluded pathogenic variants associated with NDD in this revision of the genotypic spectrum. Ethnicity data was also unavailable for the majority of NDD variants listed in these datasets therefore we were unable to explore variation in terms of genetic ancestry. This emphasizes the need for submitters of genetic variants to include as much phenotypic information as possible to aid future researchers and clinicians in their interpretation of genetic variant in association with rare diseases.

### 4.3. Considerations when assigning pathogenic scores to *CTCF* variants

From this analysis, we present an additional 86 variants including SNVs and CNVs, that have not previously been reported in the literature. The majority of pathogenic *CTCF* variants identified in association with NDD phenotypes were missense mutations affecting the protein coding sequence. We described many *CTCF* mutations which lie at well characterized regions of *CTCF*, mainly at key residues that lie within the central ZF DNA binding domain and other partially characterized functional domains including the YDF domain in the N terminus and RNA binding domains in ZF1, ZF10 and the C terminus (Nakahashi et al., 2013; Li et al., 2020; Pugacheva et al., 2020). Many of those mutations at key ZF residues are predicted to result in loss of function however it has been shown that R339Q (found in ALL and NDD) and L309P (found in ALL) in *CTCF* can result in gain-of-function phenotypes in cell growth assays (Bailey et al., 2021). Other mutation studies show the abrogation of *CTCF* binding at only select DNA binding sites, not all, supporting the idea that mutations in *CTCF* can result in a gain or change of function (Filippova et al., 2002). This remains to be explored with respect to genome-wide binding, chromatin structure and gene regulation. The pathogenicity of each *CTCF* variant was evaluated according to the AGMC guidelines and functional data from *CTCF* mutant/depletion studies. 14 nonsynonymous *CTCF* variants were reported without any pathogenicity score or listed as likely benign/benign. One variant p.Cys296Gly was reported in a proband with DD and had no pathogenicity score. However, this mutation affects the first zinc coordinating Cys residue (Figure 6). Mutations at zinc coordinating residues impairs *CTCF* binding across the genome therefore this variant was reclassified as likely pathogenic (Nakahashi et al., 2013). Many other mutations associated with NDD were identified outside of these characterized residues and domains, but their mechanism of pathogenicity remains unknown. Due to this, many of these variants remain scored as a variant of unknown significance (VUS) but it must be emphasized that despite a lack of functional data for each variant, it remains that *CTCF* is highly conserved throughout evolution and remains under mutational constraint in the human population. This should be taken into consideration when assigning pathogenicity scores of newly identified *CTCF* variants. Variants should be reviewed often and consider new experimental data. This will assist future reporting of *CTCF* variants associated with disease and continue to provide insights regarding pathogenic mechanisms. Additionally, further studies should aim to characterize variants observed in individuals with NDD that do not lie at previously characterized residues, for example, mutations that lie in the linker region between ZFs. Such efforts will help elucidate further pathogenic mechanisms of *CTCF* but perhaps also reveal a new understanding of *CTCF* function.

### 4.4. Noncoding *CTCF* variants and *CTCF* binding sites

Aside from variants affecting exons, we identified 86 noncoding sequence nucleotide variants in *CTCF*. These have not yet been reported in association with CRD however in our analysis, 31 (36%) were reported in association with an NDD phenotype. The majority of GWAS variants associated with traits or disease are identified in noncoding (intragenic/intronic) regions of the genome however the role of noncoding variants in *CTCF* has not yet been studied and deserves further attention. Whilst these noncoding variants were not included as part of the genotypic spectrum associated with NDD phenotypes, this dataset provides a resource to assist further studies. In addition to germline variants in *CTCF* being associated with neurodevelopmental disorder, genome-wide association studies (GWAS) have also identified *CTCF* variants that are associated with schizophrenia. One example shows that genetic variant rs2535629 confers risk of schizophrenia by mutating a *CTCF* binding site near the promoter of SFMBT1. This mutation impairs *CTCF* binding, causing deregulated expression of SFMBT1, a gene that plays roles in neurodevelopmental processes and synaptic morphogenesis (Li et al., 2022). It has been proposed that neurodevelopmental disorders and psychiatric disorders are exist on a spectrum, which are linked via shared molecular pathways (Morris-Rosendahl and Crocq, 2020). The role of *CTCF* in this capacity serves as an example of how its essential function in neurological processes can result in different outcomes along the neurodevelopmental continuum, with genetic variants playing a large role in its ability to function correctly. Other GWAS studies have identified noncoding SNPs within the *CTCF*s introns and 5′ UTR associated with other blood-related phenotypes, including lipoprotein levels (rs77172747) (Sinnott-Armstrong et al., 2021), eosinophil percentage of white cells (rs113028056) (Vuckovic et al., 2020) and hemoglobin concentration (rs80190634) (Sakaue et al., 2021).

### 4.5. Triplosensitivity of *CTCF* as a pathogenic mechanism underlying NDD phenotypes

Previous case reports of CNVs associated with CRD (i.e. CNVs that contain *CTCF*) have so far only described copy number losses. In this study we described an additional 29 CNVs associated with phenotypes consistent with CRD. Interestingly, we identified several instances of copy number gains being associated with phenotypes that are consistent with those reported in CRD. For example, a pathogenic 24.8 Mb copy number gain (chr16:65,347,298–90,148,393; GRCh37; ClinVar accession: VCV000058645.1) was identified in a patient with DD and other significant developmental and morphological phenotypes. This CNV was reported by Kaminsky et al., who synthesized CNVs from 15,479 individuals with DD, ID, dysmorphic features, multiple congenital anomalies, autism spectrum disorder (ASD), or clinical features suggestive of a chromosomal syndrome (Kaminsky et al., 2011), providing one of the largest CNV datasets available to date. A recent meta-analysis by Collins et al. assessed the dosage sensitivity of autosomal genes by analysis of rare CNVs associated from over 1 million human subjects across 54 disorders (including NDD) (Collins et al., 2022). Collins et al. showed that haploinsufficiency genes that are evolutionarily conserved and mutationally constrained in humans, like *CTCF*, are highly likely to be triplosensitive (i.e., duplication intolerant). Exploring the supplementary data from Collins et al., revealed *CTCF* showed bidirectional dosage sensitivity (i.e., both haploinsufficient and triplosensitive).

*In vitro*, ectopic overexpression of *CTCF* in multiple cell lines results in cell proliferation blockage, causing cell-growth inhibition, faulty DNA replication and post-mitotic cell division, demonstrating the detrimental effects of *CTCF* gains and amplifications (Rasko et al., 2001). Thereby, we propose that gain of an additional copy of *CTCF* contributes to the pathogenicity of NDD phenotypes. The effect of dysfunctional chromatin looping and gene expression during development is a growing area of research however the exact mechanisms of pathogenicity in CRD remain to be uncovered (Lupiáñez et al., 2015; Hanssen et al., 2017; Chakraborty et al., 2023). One puzzle that remains is that fast depletion of *CTCF*, using auxin-inducible degron systems in cell-based models, have not resulted in dramatic changes to enhancer-promoter interactions or transcription, highlighting a tolerance within cell assays to *CTCF* loss (Alharbi et al., 2021; Hsieh et al., 2022; Hyle et al., 2023). However, when *CTCF* is depleted *in vivo*, it does produce severe developmental phenotypes. Further work is needed to identify how *CTCF* mutants affect developmental pathways. Based on the existing literature, we propose that whilst many pathogenic germline *CTCF* variants are predicted to result in a loss of *CTCF*, certain mutations may also induce a change of function. This could result in different effects on the genome during crucial stages of development, leading to a range of impacts on chromatin organization and transcription, which may contribute to the broad spectrum of CRD/NDD phenotypes. The only functional data pertaining to NDD associated *CTCF* mutations, comes from RNA-seq in the lymphocytes from NDD patients with *CTCF* variants. It was found that in all patients carrying mutant *CTCF*, over 3000 genes were differentially expressed (compared to controls carrying no *CTCF* mutations), with the highest degree of change being found in those with frameshift mutations (Konrad et al., 2019). To date, studies investigating the impact of *CTCF* mutations on DNA binding, gene expression and chromatin structure are focused on mutations found in cancer. Similar studies to explore the impact of *CTCF* mutations found in NDD in appropriate neurobiological models have not yet been performed and should be a focus for future research. Additionally, current data exploring the impact of *CTCF* depletion in neurobiological models have been performed however no study has yet assessed the molecular impact of *CTCF* triplosensitivity, which remains another avenue to explore.

### 4.6. Conclusion

To the best of our knowledge, this is the first study that integrates genetic variant data from across multiple genotype-phenotype databases to explore the mutational spectrum of CRD. An advantage of this study is that we have provided a comprehensive and curated catalog of all *CTCF* variants known to date, which can aid diagnosis and further research efforts. We have increased the transparency of genetic variants in *CTCF* with phenotypic associations, that can be easily accessed by the clinical and research community.

## Data availability statement

The original contributions presented in the study are included in the article/Supplementary material, further inquiries can be directed to the corresponding authors.

## Author contributions

EP conceptualized the study. EP and LMF collected and analyzed data, produced the figures, and wrote the manuscript. EP, LMF, EMP, YJJ, DL, and VVL contributed to the interpretation of data and review and editing of the manuscript. VVL supervised the entire project. All authors contributed to the article and approved the submitted version.
