# Supplementary material for: An updated catalog of CTCF variants associated with neurodevelopmental disorder phenotypes
Source: Front Mol Neurosci. 2023 May 31;16:1185796. doi: 10.3389/fnmol.2023.1185796 (PMC10264798; doi:10.3389/fnmol.2023.1185796)
Supplement: Supplementary file 1 [file Data_Sheet_1.DOCX]

Supplementary Material

**An updated catalog of CTCF variants associated with neurodevelopmental disorder phenotypes**

**Emma Price^1^*, Liron Fedida^1^, Elena M Pugacheva^1^, Yon Ji^1^, Dmitri Loukinov^1^, Victor Lobanenkov^1^*.**

^1^Molecular Pathology Section, Laboratory of Immunogenetics, National Institute of Allergy and Infectious Disease, National Institutes of Health, Bethesda, MD, USA.

*** Correspondence:**Emma Price [emma.price2@nih.gov](mailto:emma.price2@nih.gov) & Victor Lobanenkov [vlobanenkov@niaid.nih.gov](mailto:vlobanenkov@niaid.nih.gov)

**Supplementary file 1.** Human phenotype ontology terms of clinical features reported in CTCF-related neurodevelopmental disorder.

**Supplementary file 2.** Single nucleotide variants in CTCF reported in genotype-phenotype databases with associated phenotypic information.

**Supplementary file 3.** Copy number variants containing CTCF reported in genotype-phenotype databases with associated phenotypic information.

**Supplementary figure 1.** Nonsynonymous sequence variants in CTCF associated with NDD. The Y axis on each plot indicates type of mutation; nonsense (green), missense (red), inframe deletion (yellow), frameshift (blue). The x axis on each plot indicates the position of each amino acid residue along the length of CTCF from the N terminus (left) to C terminus (right).

**Supplementary figure 2.** Analysis of CTCF SNPs present in the general population. (A) Allele frequencies of common CTCF SNPs identified in the 3’ UTR and ZF5 across different populations. Red line indicates allele frequency threshold of 0.05. Above 0.05 is considered common. (B) Summary of allele counts for all SNPs identified in human genomes based on mutation type. (C) Distribution of CTCF SNPs identified in general human population based on location across protein sequence.
