# Supplementary figures and images for: An updated catalog of CTCF variants associated with neurodevelopmental disorder phenotypes
Source: Front Mol Neurosci. 2023 May 31;16:1185796. doi: 10.3389/fnmol.2023.1185796 (PMC10264798; doi:10.3389/fnmol.2023.1185796)

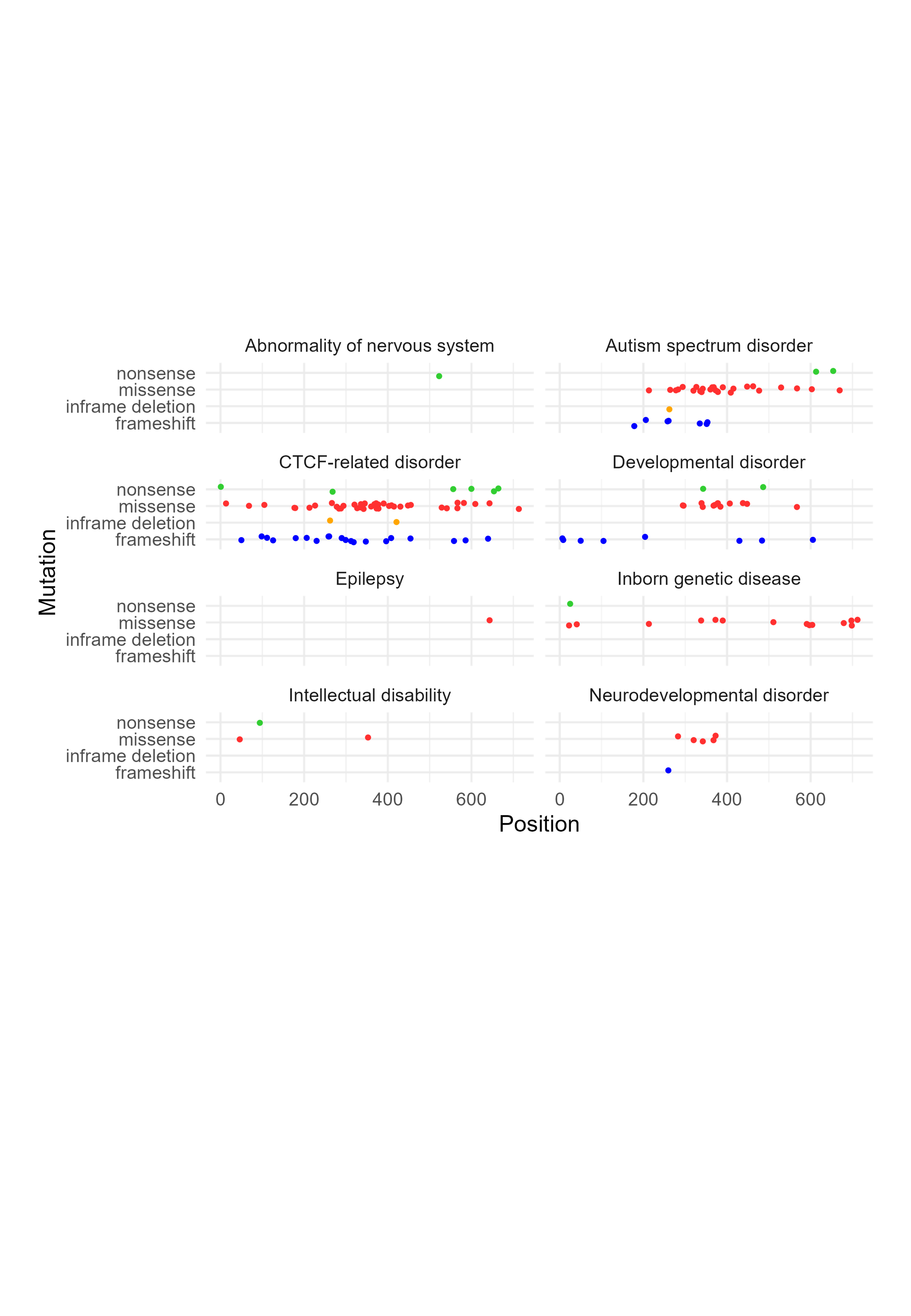

Supplement: Supplementary file 5 [file Image_1.TIF]

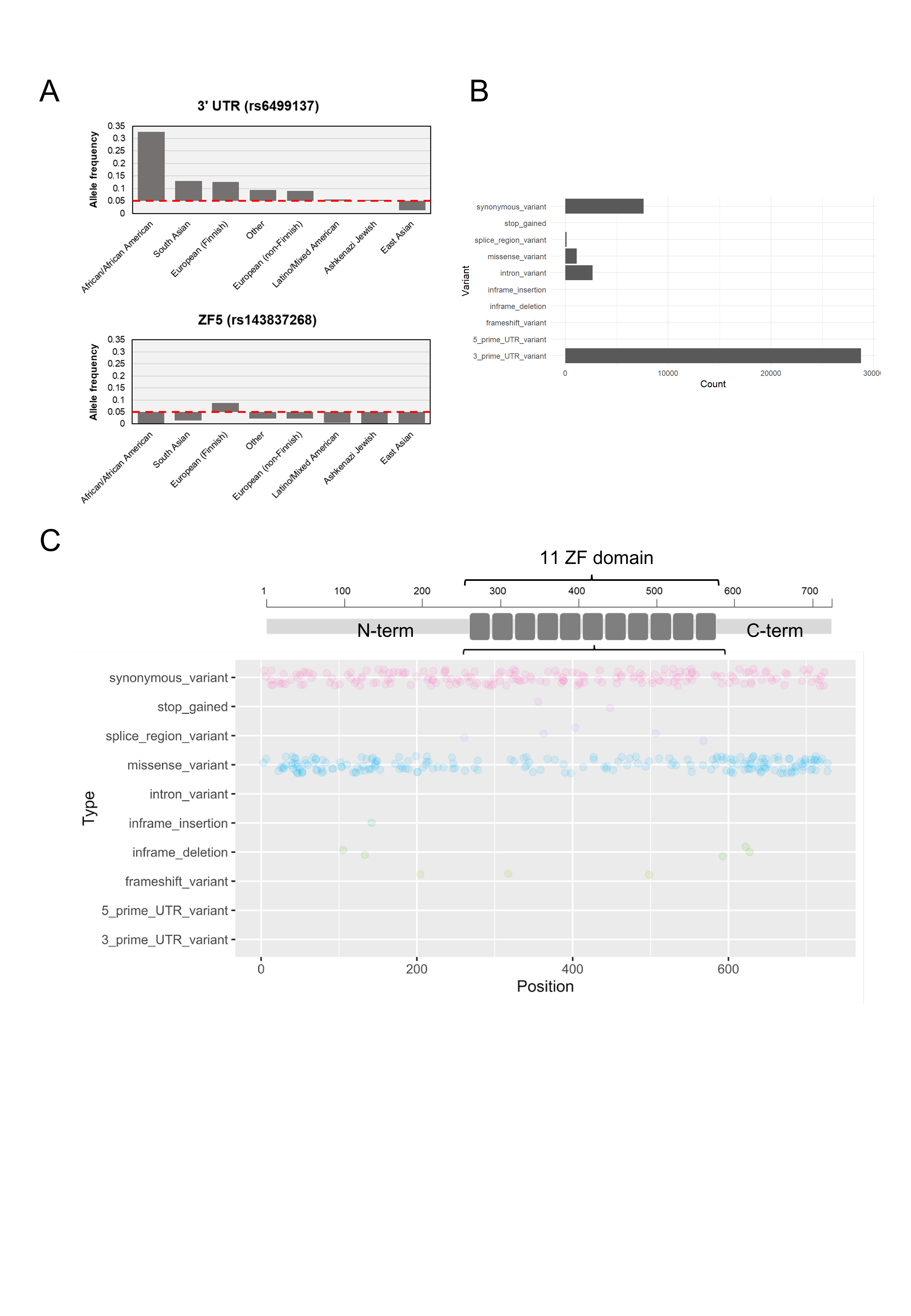

Supplement: Supplementary file 6 [file Image_2.TIF]
